# Molecular Alterations in Gastric Intestinal Metaplasia Shed Light on Alteration of Methionine Metabolism: Insight into New Diagnostic and Treatment Approaches

**DOI:** 10.3390/biomedicines13040964

**Published:** 2025-04-15

**Authors:** Nigatu Tadesse Gebrehiwot, Ying Liu, Juan Li, Hong-Min Liu

**Affiliations:** 1 School of Pharmaceutical Sciences, Institute of Drug Discovery and Development, Zhengzhou University, Zhengzhou 450001, China; znigat14@yahoo.com; 2 Key Laboratory of Advanced Drug Preparation Technologies, Zhengzhou University, Ministry of Education, Zhengzhou 450001, China; 3 The First Affiliated Hospital of Zhengzhou University, Zhengzhou 450052, China; yingv5@sina.cn

**Keywords:** gastric intestinal metaplasia, methionine, S-adenosylmethionine, PI3K/Akt/mTORC1/c-MYC, *CDX2* gene, gut microbiota

## Abstract

Gastric intestinal metaplasia (GIM) is a precancerous lesion and the key risk factor in the development of gastric cancer (GC), but early detection and treatment remain challenging. The traditional endoscopic diagnosis of metaplastic lesions is complicated by an increased rate of inappropriateness and false negativity. Although early interventions with *H. pylori* eradication, as well as endoscopic therapy results, were promising, there is still a significant unmet need to control GIM progression and recurrences. Molecular alterations, such as an increased DNA methylation index, have been identified as a crucial factor in the downregulation of tumor suppressor genes, such as the caudal-type homeobox (*CDX2*) gene, which regulates epithelial cell proliferation and GIM progression and is associated with treatment failure. *CDX2* is downregulated by promoter hypermethylation in the colonic-type epithelium, in which the methylation was correlated with reduced intake of dietary folate sources. Tumor cells alter to dietary methionine sources in the biosynthesis of S-Adenosylmethionine, a universal methyl donor for transmethylation, under the conditions of limited folate and B12 availability. The gut microbiota also exhibited a shift in microbial composition, which could influence the host’s dietary methionine metabolism. Meanwhile, activated oncogenic signaling via the PI3K/Akt/mTORC1/c-MYC pathway could promotes rewiring dietary methionine and cellular proliferation. Tumor methionine dependence is a metabolic phenotype that could be helpful in predictive screening of tumorigenesis and as a target for preventive therapy to enhance precision oncology. This review aimed to discuss the molecular alterations in GIM to shed light on the alteration of methionine metabolism, with insight into new diagnostic and treatment approaches and future research directions.

## 1. Introduction

Gastric cancer (GC) is the fifth most common and the fourth most deadly cancer in the world [1,2], with a very low rate of patient survival due to late-stage diagnosis and therapy [3]. It is a heterogeneous disease with the alteration in gut microbiota (GM) identified as the primary etiological factor in pathogenesis and prognosis [4]. The WHO, in 1974, identified *Helicobacter pylori* (*H. pylori*) bacteria as a type I carcinogen that induces gastric carcinogenesis [5]. *H. pylori* infection induces a multi-stage carcinogenesis process defined by Correa’s cascade, which includes chronic atrophic gastritis (CAG), gastric intestinal metaplasia (GIM), dysplasia, and, ultimately, adenocarcinoma sequences. In the prognosis of GC, the stage of GIM is crucial as it undergoes early tumorigenic changes, such as modifications in gut microbiota that may influence nutrient metabolism [6,7], leading to significant molecular alterations [8]. These changes are characterized by an increased DNA methylation index, marked by the downregulation of tumor suppressor genes [9] and the oncogenic expression of c-MYC downstream of mTORC1, facilitating metabolic reprogramming [10,11,12] and cellular proliferation [13].

GIM is a precancerous lesion that occurs when the lining of the gastric mucosa epithelium undergoes a histopathological transformation to resemble the intestinal cell phenotype. Unless early interventions are made to arrest the malignant progressions of GIM, the accumulation of molecular alterations increases the patient’s lifetime risk of developing or dying from GC. However, the early detection of GIM lesions through endoscopic diagnosis is complicated by an increased rate of inappropriateness and false negativity. The strength of the challenge indicates that specific and sensitive diagnostic biomarkers are highly required. Moreover, endoscopic surgical treatment is considered only in those with clearly identified lesions. As a result, early intervention with mass eradication therapy targeting *H. pylori* is recommended [14]. However, since only a small proportion (0.25–2.5%) of GIM progress to GC, surveillance and prevention approaches have varied across several regions [15]. The mass eradication of *H. pylori* is mostly confined to the high-risk East Asian countries such as China, Korea, and Japan, which have implemented screening and *H. pylori* eradication programs as an early prevention strategy [16,17,18,19], whereas the Western countries typically limited the screening and follow-up to the high-risk individuals [20]. Although *H. pylori* eradication therapy is indicated as a promising therapy to limit the risk of GIM progression, existing study reports remained controversial regarding complete GIM reversal [21]. This has led to the conclusion that other factors needed to be evaluated regarding to a potential role in GIM progression [22] and recurrences [23].

Studies have indicated that the ectopic expression of the tumor suppressor caudal-type homeobox (CDX2) gene is involved in the differentiation of gastric mucosal epithelial cells into intestinal cell phenotypes, and the subsequent downregulation promoted cellular growth, proliferation, and GIM progression, which thereby influenced treatment efficacy [24,25,26,27]. A shift in gut microbiota composition has been also reported in the progression of GIM, which could interact with methionine metabolism to modulate residual DNA methylation [28]. Tumor cells exhibited methionine dependence to satisfy transmethylation demands in the context of folate and B12 deficiency. *CDX2* gene methylation increased with the patient’s history of reduced dietary folate intake [25], indicating the potential role of a metabolic alteration in methionine metabolism. Altered methionine metabolism could be exploited as a diagnostic metabolic marker of tumorigenesis and a targete for preventive therapy to enhance precision oncology [29,30]. This article aimed to review the molecular alterations in GIM progression that shed light on the alteration of methionine metabolism, with insight into new diagnostic and treatment approaches and future research directions.

## 2. Gastric Intestinal Metaplasia

Gastric intestinal metaplasia (GIM) manifests as the transformation of atrophic gastritis mucosa cells into intestinal epithelial cells [31]. The early development exhibited a gradual change resembling a phenotype of intestinal epithelial cells, known as complete GIM, alongside an incomplete GIM type characterized by a mixture of gastric- and colonic-type epithelial cells; nevertheless, the chronology of occurrence remained controversial [32]. In the prognosis of GC, the GIM was identified with significant early molecular alterations, though the relevance remained poorly understood. GIM increased the risk of developing GC by 3.5%, which increases toward incomplete GIM [33]. A multivariate analysis conducted in Spain, with a mean follow-up of 12.8 years, detected an incidence of GC in 16.0 (18.2%) out of 88 cases with incomplete GIM and only in 1 (0.96%) out of 104 patients with complete GIM (HR 11.3, 95% CI: 3.8–33.9) [34]. In another long-term surveillance study in the UK, a yearly endoscopic follow-up detected stage I GC in 36% of patients with GIM [35], and a similar rate of 38% was achieved in Italy, with two years of endoscopic follow-up [36]. Thus, GIM could represent a significant risk for developing GC, and early follow-up and treatment could increase the likelihood of early detection and prevention [37]. Treatment modalities for GIM mainly include endoscopic surgical therapy, but they are challenged with detecting residual metaplastic lesions [38], which is also attributed to the incidence and recurrence of interval early GC [23]. Furthermore, early intervention with *H. pylori* eradication therapy was indicated to be promising, yet complete GIM reversal remained a controversy [21,22]. Thus, the early detection and treatment of GIM involve a clear unmet need in gastroenterology.

GIM is identified with significant early molecular alterations characterized by a slower rate of progression (6.1 latent years) toward GC, which could give a window period of opportunity for proper exploration [7,39]. Among the various molecular alterations identified in GIM, the downregulation of the caudal type homeobox-2 (*CDX2*) gene plays a significant role in GIM progression to dysplasia and GC [27]. The downregulation of the *CDX2* gene occurs via promoter hypermethylation and was correlated with the patient’s history of reduced folate sources intake [25]. Tumor cells depend on dietary methionine as a mechanism to satisfy the demand for an increased rate of gene transmethylation and to promote cellular growth and proliferation [29]. Currently, targeting dietary methionine is considered a promising strategy to limit tumor cell growth, proliferation, and progression, as well as to predict tumorigenesis as a strategy to enhance precision oncology [29,30].

## 3. Molecular Alterations to Shed Light on Altered Methionine Metabolism

The accumulated study evidence indicated preliminary tumorigenic changes in the tumor microenvironment (TME) of GIM, such as a shift in the gut microbiota composition with a relative abundance of specific bacterial species that could interact with the host methionine metabolism to influence DNA methylation, which could enhance tumorigenesis and progression [40,41]. Accordingly, the change in gut microbiota could be co-characterized with accompanied significant molecular alterations during GIM progression that could shed light on the alteration of methionine metabolism, such as an increased DNA methylation index marked by the downregulation of several tumor suppressor genes [9], as well as upregulations in oncogenic signaling via the PI3K/Akt/mTORC1/c-MYC pathway to facilitate the metabolic rewiring of dietary methionine [10,11,12], and promote cellular proliferation [13].

### 3.1. Gut Microbiota Alteration

The gut microbiota interacts with nutrient metabolism to alter metabolites that can contribute to the tumorigenesis and prognosis of cancer [42,43]. Several studies indicated that the change in the profile of gut microbiome composition is associated with the upregulation of different metabolic pathways and is identified as a risk factor for reduced treatment efficacy in GC [44]. Thus, the development of diagnostic and therapeutic agents in GC should consider the role of gut microbiota alteration in nutrient metabolism. Altered gut microbiota could modulate folate and methionine metabolism to influence epigenetic mechanisms during gastric carcinogenesis [6,45,46]. The gut microbiota’s role in methionine metabolism could be crucial in regard to the downregulation of the CDX2 gene via promoter hypermethylation, which is correlated with a patient’s dietary history. Thus, it is imperative to investigate the profile of gut microbiota composition with regard to the role of the perturbation of nutrient metabolism in GIM. Studies indicated that the abundance of *H. pylori,* as the primary carcinogen, was negatively correlated with both folate and methionine metabolism but showed a decreasing trend from CAG to GIM, dysplasia, and GC with a concomitant increasing trend in the abundance of *lactobacillus* [47]. More importantly, *lactobacillus* strains were identified to promote folate biosynthesis and positively correlated with methionine metabolism [48]. Hence, it is crucial to explore the impact of the relative abundance of the two bacterial strains on dietary methionine metabolism in GIM.

#### 3.1.1. Helicobacter Pylori Abundance

The normal gut microbiome is composed mainly of the bacterial phyla *Firmicute* (with the genera *Lactobacillus*, *Clostridium*, *Bacillus*, *Enterococcus*, and *Ruminicoccus*) and Bacteroides, which ccount for more than 90%, followed by proteobacteria, which include the pathogenic bacteria genus *H. pylori* [49]. The early stage of gastric carcinogenesis is characterized by a relative abundance of *H. pylori* [50]. A study on the interaction of gut microbiota and metabolites in GC demonstrated a negative correlation between *H. pylori* abundance and amino acid biosynthesis, such as methionine [48]. Basically, gastric mucosa cells can synthesize methionine de novo through Hcy remethylation in the one-carbon metabolism pathway. However, the depletion of folate and vitamin B12 (VB12) reduces Hcy remethylation in AG [51]. Folate is a methyl group donor, and VB12 acts as a co-factor of the methionine synthase (MS) enzyme to transfer the methyl group to Hcy during the remethylation process [52]. *H. pylori* infection impairs nutrient metabolism through several complicated mechanisms [53].

**Figure 1:**
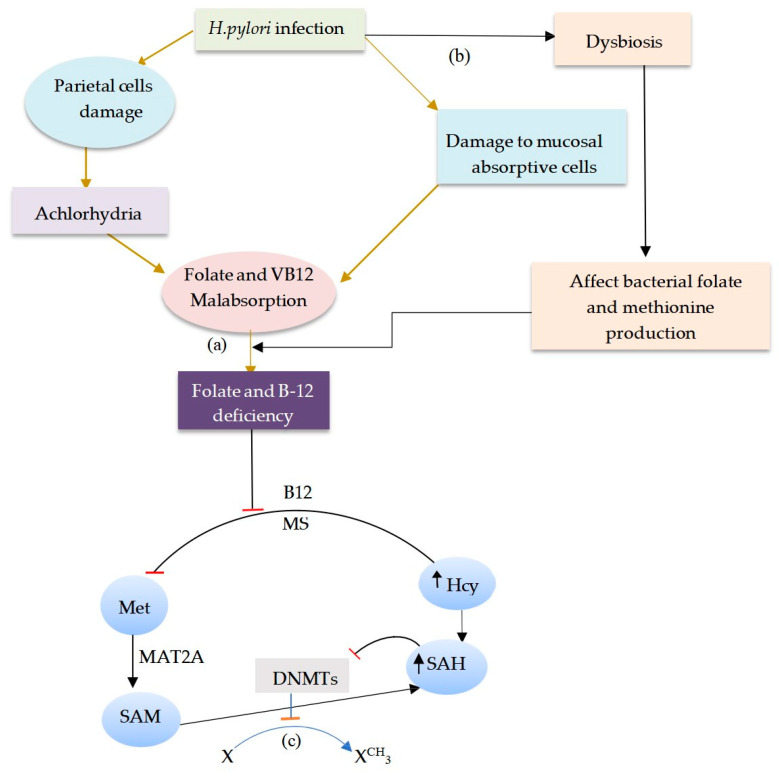
The role of *Helicobacter pylori* infection in methionine metabolism. *H. pylori* is associated with a risk of folate and VB12 deficiencies (**a**), leading to impaired methionine regeneration in the 1C metabolism pathway. *H. pylori* can also induce gut microbiota dysbiosis and impaired bacterial-induced folate and methionine production (**b**). Dysregulation of methionine metabolism in the 1C metabolism pathway contributes to the occurrence of global DNA hypomethylation due to the increased Hcy level, which converts back to SAH, an allosteric inhibitor of DNMTs, to impair DNA methylation (**c**). The risk of global hypomethylation due to nutrient deficiencies in AG was evident in a study’s findings that demonstrated a high rate of methyl carbon incorporation, whereas the rate of incorporation decreased to become constant in GIM and dysplasia [65], which suggested the likelihood of methyl donor metabolism in GIM. MS: methionine synthase; SAM: S-Adenosylmethionine; SAH: S-Adenosylhomocysteine; Met: methionine; DNMT: DNA methyltransferase; Hcy: homocysteine; GH: global DNA hypomethylation; 5-MTHF: 5-methyl tetrahydrofolate. Red blocked arrow: blockage. Pointed arrow: process.

*H. pylori* infection-induced inflammation causes damage to gastric mucosa absorptive cells through atrophy and loss of digestive glands [54], which impairs the absorptive functions of the mucosal epithelium [54]. Moreover, the inflammation cross-talk via interleukin-1β (IL-1β) on parietal cells induces HK-ATPase enzyme dysfunction and inhibits the production of stomach acid, thereby resulting in a condition also known as achlorhydria [55,56]. Studies indicated that the absorption of folate and VB12 could be impaired in the low acidity of the stomach [53,57]. For instance, low acidity accounted for impaired absorption of VB12 mainly by affecting the dissociation from the carrier protein [58]. Moreover, *H. pylori* colonization also contributes to a reduction in commensal bacteria abundances, such as *Bifidobacteria* and *Lactobacillus*, as the well-known producers of folate [59,60,61], resulting in dysbiosis. The depletion of folate and VB12 is attributed to the risk of Hyperhomocysteinemia (HHcy) [62], a common phenomenon in GC as well as in gastric precancerous lesions [63] (Figure 1). However, HHcy can also occur due to dietary methionine metabolism [64], which should be differentiated from HHcy due to impaired folate and B12 metabolism. Dietary methionine metabolism is unlikely to occur in *H. pylori*-predominant mucosa due to the risk of gut microbiota dysbiosis [53]. This implies that the abundance of *H. pylori* could impair both endogenous and dietary methionine metabolism in CAG. However, interestingly, as the relative abundance of *H. pylori* diminishes in the progression of AG to GIM and dysplasia toward GC, the increasing relative abundance of Gram-positive bacteria, such as lactobacillus, increases, suggesting the need to explore the potential impact on methionine metabolism.

#### 3.1.2. Prevailing Abundance of Lactobacillus

**Figure 2:**
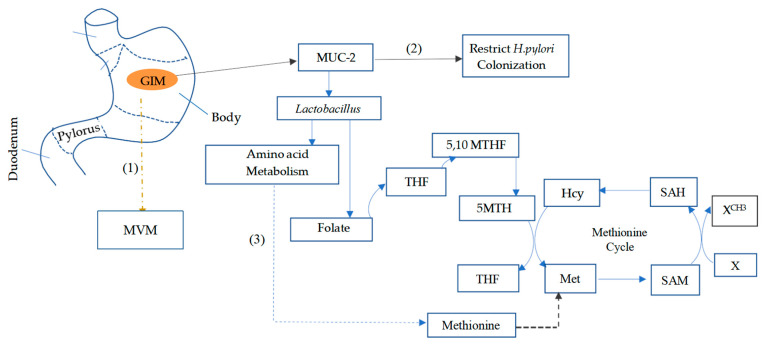
Schematics of gut microbiota shifts in gastric intestinal metaplasia and the impact on methionine metabolism. (1) GIM constitutes a change in morphological features through developing the intestinal epithelial cell phenotype with well-defined enterocyte absorptive microvilli, replacing damaged atrophic mucosa cells, crucial for nutrient absorption. (2) The relative abundance of lactobacillus induces the expression of MUC2 mucin, a type of mucin that can restrict *H. pylori* colonization. As a result, *H. pylori* tests often become negative at this stage. (3) *Lactobacillus* are known folate producers, and their abundance is also associated with the upregulation of amino acid metabolism, including methionine, as the essential substrate to the folate and methionine cycle, respectively. GC: gastric cancer; GIM: gastric intestinal metaplasia; 5,10 MTHF: 5,10 methylene tetrahydrofolate; 5MTHF: 5-methyl tetrahydrofolate; Hcy: homocysteine; SAM: S-Adenosylmethionine; SAH: S-Adenosylhomocysteine; X: methyl group acceptors, such as DNA, RNA, and histone. MVM: microvillar membrane crucial for nutrients absorption, Brown broken arrow: development of well-defined enterocyte absorptive microvilli. Blue broken arrow: bacteria-induced dietary methionine production. Black broken arrow: dietary methionine metabolism.

*H. pylori* infection faces suicidal mucosal colonization during the progression of AG to GIM. *H. pylori* mainly colonizes the gastric mucosa by binding onto the mucin glycoprotein (MUC1) through fucosylation and sialylation via glycan [66,67]. However, *H. pylori* colonization depletes MUC1 by decreasing the rate of synthesis [68]. Subsequently, MUC1 changes to the MUC2 phenotype, which restricts *H. pylori* colonization [69] (Figure 2). In addition, the achlorhydria condition induced by *H. pylori* also enhances the development of GIM [70], maybe by promoting the overgrowth of Gram-positive bacteria abundant in the upper small intestine [71]. A trend of increased colonization by intestinal flora, such as the bacteria genus *lactobacillus,* was discovered in GIM toward GC [72,73]. Microbial network analyses also indicated the co-occurrence and a gradual increase in the abundance of *lactobacillus* in GIM toward dysplasia and GC [74,75]. A previous study on microbiota analysis of fecal samples also revealed an increasing trend in the abundance of the bacterial family *Firmicute* (mainly of the bacteria genus *lactobacillus*) in a GIM group (20.07%) compared to AG (7.23%) and normal mucosa (5%) [76]. Furthermore, the evenness and diversity of the gut microbiota were reportedly increased from AG to GIM and GC. Accordingly, a complex gut microbiota dominated by other bacteria genera than *H. pylori* accelerated the development of gastrointestinal neoplasia (GIN), as demonstrated in a study on insulin–gastrin transgenic mice [77]. An analysis of the contribution of individual bacteria genera in the complex to the development of GIN, using a restricted commensal microbiota known as Altered Schadler’s flora (ASF), identified that the overgrowth of *lactobacillus murinus* ASF361 promoted the development of neoplasia in the same way as normal intestinal flora [78], suggesting the potency of *lactobacillus* abundance on GIM progression. *Lactobacillus* strains could contribute to the development of neoplasia via diverse mechanisms. Some *lactobacillus* strains have probiotic effects such as improving the gut micro-ecology by promoting the gut mucosal barrier and inducing *MUC2* mucin gene expression [79,80,81]. In contrast, other *lactobacillus* strains could interact with the host’s nutrient metabolism by promoting the degradation of dietary proteins to produce amino acids [82]. A recent study on the interaction between *lactobacillus* abundance and different metabolic pathways in GC indicated a positive correlation with amino acid biosynthesis, including methionine [48] (Figure 2). However, limited studies are currently available on the role of the relatively increasing abundance of lactobacillus on dietary methionine metabolism in GIM.

### 3.2. Increased Gene Methylation Index and CDX2 Downregulation

Epigenetic silencing of genes by aberrant DNA methylation is the crucial mechanism contributing to the tumorigenesis and prognosis of GC. Residual DNA methylation is strongly associated with the progression of GIM to GC [28], characterized by a higher gene methylation index with the downregulation of several tumor suppressor genes compared to superficial gastritis and CAG [9,83,84]. The downregulation of the tumor suppressor caudal type homeobox 2 (*CDX2*) gene regulates the transdifferentiation of gastric mucosa epithelium into the intestinal epithelial cell morphological phenotype and is considered an intestinal specific transcription factor (TF) gene responsible for the coexpression of intestinal markers, such as the Mucin 2 (*MUC2*) gene [85], implying a role in the development of GIM from CAG. Although the expression of the *CDX2* gene promotes intestinal-type cell differentiation, its downregulation is associated with increased epithelial cell proliferation [24]. Studies indicated that incomplete GIM with a colonic type columnar epithelium was identified as prevalent toward dysplasia and GC, characterized by a reduced expression of intestinal markers, such as MUC 2 type mucin [86], and a concurrent progressive downregulation of the *CDX2* gene [27]. The progressive downregulation of the *CDX2* gene was also identified as a prognostic marker and determinant of treatment inefficacy in GC [87]. A previous study demonstrated that the *CDX2* gene was downregulated in colonic-type gastric epithelial cells through promoter region hypermethylation, which exhibited a relationship of increased methylation with a patient history of dietary folate intake, such as reduced consumption of green tea and cruciferous vegetables [26], suggesting the need to explore the potential role of metabolic alteration in methionine metabolism.

### 3.3. Cellular Proliferation

Tumor cell proliferation depends on nutrient availability, such as amino acids [88]. Methionine is an essential amino acid to serves as a precursor of SAM biosynthesis that acts as a universal methyl donor of transmethylation to promote protein synthesis and cell proliferation [89]. Accordingly, cancer cells exhibited methionine auxotrophy and subsequent reprogramming to dietary methionine sources [29]. Methionine is converted intracellularly to SAM, catalyzed by methionine adenosyl transferase 2A (*MAT2A*) in the cytoplasm, and acts as a methyl donor for cellular methylation. Hoffman and colleagues demonstrated that although methionine-dependent tumor cells could synthesize a normal amount of methionine endogenously, the biosynthesis of SAM was insufficient because of the increasing demand for transmethylation [90]. SAM enhances cell cycle progression and proliferation by promoting maintenance methylation during DNA synthesis at the G1 phase. However, the effect of SAM depends on its intracellular concentration [91], with a low intracellular SAM levels could induce cell cycle arrest at the G1 phase [92]. For instance, a previous study indicated that an increased intracellular content of SAM relative to S-Adenosylhomocysteine (SAH) promoted the growth of adenocarcinoma 755 and sarcoma 180 of experimental tissue origins [93]. An increased SAM level was also identified in response to cellular proliferation activity in cultured lymphoblasts [94]. Moreover, there is also a theory which stated that the GIM be developed from bone-marrow-derived stem cells (BMDSCs) [95], in which the stem cells nature renders a unique characteristics of higher methionine requirement for SAM biosynthesis to promote cell proliferation and tumor progression [96]. Accordingly, studies also indicated increased cell proliferation in GIM with reduced apoptosis potential [13], suggesting the likelihood of SAM biosynthesis. Interestingly, an increased intracellular content of SAM in tumor cells could represent a differential methionine cycle activity in response to exogenous methionine metabolism compared to normal cells that depend on the folate cycle for de novo synthesis of methionine via Hcy remethylation [90]. Hence, the increased cellular proliferation in GIM could be suggestive of a higher methionine demand for SAM biosynthesis, which should be evaluated in the context of potential alteration to dietary methionine sources.

#### The PI3K-Akt-mTORC1 Signaling Pathway in Cellular Proliferation

**Figure 3:**
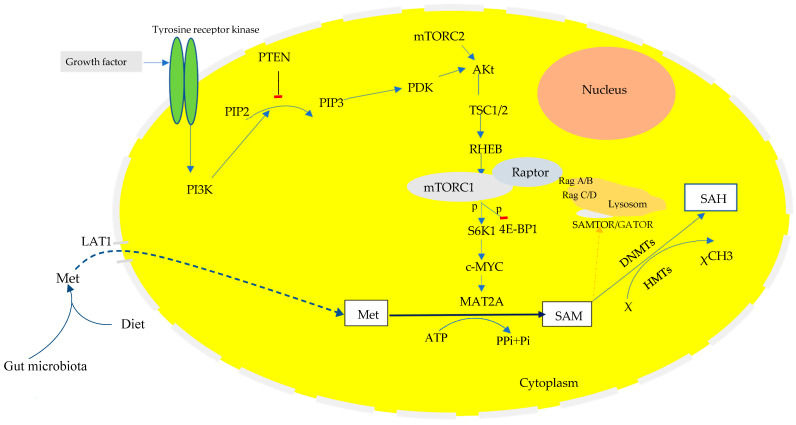
The role of the activated PI3K/Akt/mTOR/c-MYC pathway in metabolic reprogramming to dietary methionine sources. mTORC1 is an anabolic enzyme activated by amino acid metabolism and promotes the oncogenic expression of c-MYC to enhance metabolic reprogramming and drive tumorigenesis. Altered gut microbiota with relative abundance of LB over HP is a phenomenon in GIM and GC, in which the interaction between LB and nutrients is associated with the upregulation of amino acid biosynthesis, such as methionine. Growth factors such as insulin could induce the tyrosine receptor kinase-dependent activation of the PI3K/Akt/mTORC1 signaling pathway to promote the overexpression of the c-MYC oncogene in tumor cells, including in GIM. Overexpression of c-MYC promotes metabolic rewiring of exogenous methionine through two mechanisms: (1) activating LAT1 transporters and (2) promoting the transcription of MAT2A through binding to intron 1 to increase *MAT2A* expression. MAT2A catalyzes the conversion of exogenous methionine to SAM, a universal methyl donor for the methylation of tumor suppressor genes, and promotes cellular proliferation. The *CDX2* gene has a tumor-suppressor role and is downregulated with the activation of mTORC1 and the suppression of *PTEN*. The *CDX2* gene is downregulated in colonic-type epithelium via promoter region hypermethylation, which increases with reduced intake of dietary folate sources, suggesting altered methionine metabolism. mTORC11/2: mammalian rapamycin complex-1 or 2 target; Raptor: regulatory-associated protein of mTOR; SAMTOR: S-Adenosylmethionine-sensing protein of mTOR; *CDX2*: caudal homeobox type-2; tyrosine receptor kinase (TRK); LAT-1: L-type amino acid; GF: growth factor; PIP2: phosphatidylinositol-4,5-diphosphate; PIP3: phosphatidylinositol-3,4,5-triphosphate; *PTEN*: phosphatase tensin homolog; TSC1/2: Tuberous sclerosis 1/2; RHEB: Ras homolog enriched in brain; *MAT2A*: methionine adenosyltransferase 2A; c-MYC: Avian myelocytomatosis viral oncogene homolog; SAM: S-Adenosylmethionine; SAH: S-Adenosylhomocysteine; Met: methionine; DNMT: DNA methyltransferase; HMT: histone methyltransferase. Blue broken arrow: dietary methionine rewiring. Blue solid arrows: process.

Studies indicated that mitogenic activation by the mammalian target of rapamycin complex 1 (mTORC1), as the major oncogenic signaling pathway, promotes cell proliferation and tumor progression by inducing the protein translation of cell cycle control genes, such as cyclin D1, an allosteric activator of the cyclin-dependent kinases (CDK4/6), for cell cycle progression [97]. mTORC1 is activated by several factors including growth factors signals and amino acids [98,99]. Growth factors act as the ligand on the receptor tyrosine kinase (RTK), which is activated to recruit phosphatidylinositol 4,5-bisphosphate (PIP2) and is converted to phosphatidylinositol-3,4,5-triphosphate (PIP3), catalyzed by phosphatidylinositol-3 kinase (PI3K). PIP3 activates phosphoinositide-dependent protein kinase 1 (PDK1), which phosphorylates and activates Akt (also known as protein kinase B), a critical kinase at the center of the interplay for metabolic reprogramming during cellular catabolic and anabolic switch in response to nutrient status [100] (Figure 3). 

Studies indicated that, in the case of nutrient deficiency, the adenosine monophosphate kinase (AMPK) pathway is activated to phosphorylate the tuberous sclerosis complex2 (TSC2), which has GTPase-activating protein (GAP) activity, to deactivate mTORC1 by inhibiting the active Ras homolog enriched in the brain (RHEB-GTP) for cellular entry to catabolism and energy saving mode [101]. Liver kinase B1 (LKB1) is a master kinase that regulates cell metabolism through phosphorylation and activation of AMPK to downregulate the RAS activation signaling pathway, which activates the downstream PI3K/Akt/mTORC1 signaling [102]. Loss of LKB1 drives preneoplastic program in colonic-type cancers [103]. 

On the other hand, in the presence of amino acids, the growth factors activate RTK and the downstream PI3K-Akt pathway to promote phosphorylation and inhibition of the TSC1/TSC2 gene (hamartin/tuberin) which is the negative regulator of RHEB-GTP (see Figure 3). The conversion of GDP to GTP status activates RHEB binding to the mTORC1 through the nutrients sensing Raptor protein subunit. This process occurs only in the presence of amino acid binding to the regulator complex proteins of Rag GTPase.

Studies indicated that the activation of mTORC1 signaling becomes increasingly dependent on amino acids, including methionine, to promote oncogene translation during neoplastic transformation [104]. The activation of the PI3K/Akt/mTORC1 pathway is directly associated with epithelial cell proliferation [105] and has been discovered as important in the complex process for the downregulation of the *CDX2* gene [106]. Although studies indicated that *H. pylori* can promote the upregulation of the Serine/threonine-protein kinase (PLK1)/PI3K/Akt pathway to stimulate epithelial cell proliferation [107], the abundance of *H-pylori* becomes diminished in GIM and dysplasia toward GC compared to AG [73]. Moreover, *H. pylori* can also promote the expression of the CDX2 gene and the development of complete GIM through the production of the CagA protein [108]. However, *H-pylori* depletes host cell mitochondria amino acid metabolism in a vacuolating cytotoxin (VacA)-dependent manner to inhibit mTORC1 [109]. Thus, the progression of GIM, despite the diminished abundance of *H. pylori*, could be attributed to the possible occurrence of a reverse process (amino acid replenishment) to promote the activation of mTORC1. 

Furthermore, there is strong evidence of the association between mTORC1 activity and the downregulation of the CDX2 gene in the progression of GIM. A study evaluating the effect of the intestinal cell kinase (ICK) protein on CDX2 gene expression demonstrated that the expression of ICK and mTORC1 activities antagonized CDX2 gene expression. For instance, the CDX2 gene was downregulated with the upregulation of mTORC1 [110] and was overexpressed with the upregulation of the phosphatase and tensin homolog (PTEN) gene, which antagonizes the tri-phosphorylation of PIP2 by the PI3K enzyme, for the downstream activation of mTOR kinase [111]. *PTEN* expression is progressively downregulated from GIM to dysplasia and GC [112]. This suggests the role of the PI3K/Akt/mTORC1 pathway in the progressive downregulation of the CDX2 gene and cellular proliferation during gastric carcinogenesis from incomplete GIM to dysplasia and GC [113].

### 3.4. The PI3K/Akt/mTORC1-c-MYC Signaling Pathway in Metabolic Reprogramming

Tumor cells alter dietary methionine metabolism due to the increased rate of transmethylation and promote cellular growth and proliferation. Upregulations in the PI3K/Akt/mTORC1 signaling pathway regulate the expression of oncogenes, such as the Avian myelocytomatosis viral oncogene homolog (c-MYC), during gastric carcinogenesis [11]. c-MYC plays a tumorigenic role in global cellular metabolic reprogramming, cell cycle progression, differentiation, and apoptosis [114,115,116]. c-MYC also activates the L-type amino acid transporter-1 (LAT-1) to promote amino acid transport across tumor cell membranes [117]. The expression of LAT1 was identified as a prognostic marker in the progression of gastric carcinogenesis [118]. Increasing evidence indicates a link between the expression of LAT1 and the activation of mTORC1 [119]. LAT1, also known as the solute carrier 7A5 (SLC7A5), is a light-chain heteromeric amino acid transporter of neutral essential amino acids, including methionine on the cancer cell membrane, and its suppression inhibits mTORC1 activation through limiting the intracellular level of amino acids [120,121]. Amino acid metabolism regulates mTORC1 activation [122], thereby to promote the protein expression of oncogenes [123]. Amino acid metabolism activates mTORC1 either through binding at the taste-1-receptor member 1 (T1R1) and taste 1 receptor member 3 (T1R3) hetero-dimer cell-surface G-protein-coupled receptors, as a luminal sensor of amino acids extracellularly [124], or the amino acids transported across the tumor cell membrane into the epithelium for intracellular binding to their specific binding site on the regulator-Rag complex, except for cytoplasmic methionine, which has to be converted to SAM to bind to the SAMTOR/GATOR1 regulator protein complex to activate mTORC1 [122,125]. The activation of mTORC1 promotes the expression of the c-MYC oncogene through phosphorylation of the ribosomal RNA translation regulator 70s S6K and the eukaryotic translation initiation factor 4E-binding protein-1 (4E-BP1), a master regulator, to control protein synthesis by negatively regulating the eukaryotic translation initiation factor 4E (eIF4E) [11] (Figure 3).

Studies indicated that a slightly increased expression of c-MYC compared to the normal level enhanced tumorigenesis [126,127]. c-MYC was identified over expressed in GIM and GC [10,128] and could promote the transcription of MAT2A that catalyzes the conversion of dietary methionine to SAM [129]. Accordingly, MAT2A expression also identified in several extra-hepatic tissues, including colon, gastric, and pancreatic cancers [130]. The expression of MAT2A increased in tumor cells with SAM insufficiency. Hoffman and colleagues demonstrated that tumor cells exhibited SAM insufficiency due to the high rate of transmethylation [90]. Since MAT2A is the rate-limiting enzyme with a shorter half-life due to proteasomal degradation, the protein level of MAT2A should be maintained through transcriptional and post-transcription regulations [130,131], suggesting an insufficient intratumoral SAM level [132]. Thus, the oncogenic expression of c-MYC downstream of the mTORC1 pathway could play a role in tumor cell reprogramming to dietary methionine sources through promoting the transcription of MAT2A [12,133].

As mentioned above, the downregulation of the CDX2 gene in colonic-type epithelium occurs through promoter hypermethylation, with a higher rate of promoter region methylation identified in (78.5%) of colorectal cancer cells (CRCs) compared to (43.5%) healthy controls [26]. Moreover, CDX2 gene promoter methylation correlates with the patient’s history of impaired dietary folate intake [25], implying the potential of alteration in methionine metabolism. However, further studies are urgently required in GIM to uncover the role of the activated PI3K/Akt/mTORC1-c-MYC pathway in metabolic reprogramming to dietary methionine.

## 4. Insight into New Diagnostic and Treatment Approaches

As the survival from GC remains generally low, with most patients progressing to late-stage GC, early identification of precancerous lesions and therapeutic intervention remains the primary recommendation [38]. The early diagnosis and follow-up of GIM and dysplasia cases were indicated as promising in identifying prospective GC patients. However, the traditional endoscopic screening and diagnostic methods are challenged with a considerable rate of false negativity (4.6–25.8%) [134], as well as inappropriateness (22%) [135]. Currently, the value of the ratio of pepsinogen I to pepsinogen II (PGI/PGII) (less than 3), coupled with elevated gastrin-17 and ant-*H. pylori* antibodies, also used as an indicator of AG, as well as the presence of precancerous lesions [136,137]. However, the specificity and sensitivity are limited by several conditions, such as the location of *H. pylori* infection (antral type characterized by increased acidity compared to corpus region) [138], hypergastrinemia [139], or the use of acid-suppressing agents [140,141]. Thus, the screening prediction of GIM progression remains a clinical challenge [15], requiring more specific and sensitive biomarkers. Moreover, therapeutic intervention with endoscopic mucosal resection (EMR) or surgical dissection (SD) therapy are also considered only in patients with clearly identified GIM lesions [38]. However, there is still a risk of recurrence and incidence of interval early GC post-surgical therapies [23]. In addition, the strategy of early eradication therapy for *H. pylori* infection also has been affected by the regional variability in implementation and the controversies toward GIM reversibility. Thus, there is a clear unmet need in GIM for alternative diagnostic biomarkers and treatment modalities.

Studies indicated that the gut microbiota is a residual factor affecting gastric tumor progression and treatment efficacy [44]. The gut microbiota can modulate methionine metabolism to promote DNA methylation [28]. Accordingly, both the gut microbiota and residual DNA methylation have been ascribed for the progression of GIM post-*H-pylori* eradication therapy as well [142,143]. The composition of the gut microbiota with increased lactobacillus and decreased *H-pylori* abundance contributed to the host’s methionine metabolism and increased gene methylation in the progression of GIM toward GC [6,144]. Tumor cells resort to dietary methionine due to increased demand for SAM biosynthesis as a methyl donor for an increased rate of transmethylation [29]. The downregulation of the CDX2 gene through promoter DNA hypermethylation increased with the patient’s history of impaired dietary folate intake, suggesting the need to explore alteration in methionine metabolism and the potential for therapeutic exploitation.

The methionine addiction of tumor cells could be exploited as a metabolic phenotype for predictive screening of tumorigenesis and as the metabolic target for the preventive therapy to promote precision medicine [30]. For instance, in methionine-dependent tumors, the circulating level of methionine is lower compared to healthy individuals [145]. In contrast, both the production and circulating levels of SAM become elevated, which could be evaluated for a potential early diagnostic biomarker role in neoplastic progression and recurrences [146,147].

Pre-clinical studies by Hoffman and colleagues indicated that methionine restriction (MR) in methionine-dependent tumor cells reduced the relative intracellular SAM to SAH level and slowed down cellular growth, proliferation, and tumor progression [90]. In another study, methionine deficiency was identified to induce mitophagy and attenuate the migration and invasion of GC cell lines [148,149]. Moreover, dietary MR, by reducing more than 80% of the daily intake, also attenuated tumor progressions in both mouse and human models [150]. Targeting tumor cells’ addiction to dietary methionine using novel recombinant methioninase (rMETase), either alone or in combination with other anti-tumor agents was also demonstrated to be effective and safe in limiting tumor progression in human cancer cell lines (including GC) as well as animal models [29]. Recently, small molecule inhibitors are also being evaluated at early clinical development stages to target methionine metabolism in tumor cells with novel mechanisms of action, such as through the inhibition of MAT2A [133].

## 5. Future Research Directions

Advances in metabolomics have revolutionized the diagnosis and treatment of cancer and other diseases [151]. Metabolomics is one of the powerful omics techniques used to study the alteration in cellular metabolites affected during neoplastic progression [152]. Metabolomics has identified several metabolites that are dysregulated in GC and precancerous conditions [153]. The gut microbiota was discovered to modulate host methionine metabolism [6]. The advent of metagenomics using the 16s rRNA sequencing technique also enabled the profiling of gut microbiota changes and their interaction with nutrient metabolites in cancer [154,155]. The metabolomics characterization of 150 different metabolites in GC, coupled with 16s rRNA sequencing of the gut microbiota, revealed a strong correlation between the abundance of *lactobacillus* and metabolites of the methionine cycle and trans-sulfuration pathway, such as SAM, SAH, L-Cystathionine, and glutathione (GSH) [48], where a similar alteration in the gut microbiota composition is also marked in GIM. Moreover, GIM is more related to GC regarding amino acid metabolism [153]. However, so far, studies on gastric carcinogenesis have focused mostly on the change in gut microbiota composition, while the interaction and correlation with specific metabolites have not yet been studied. Thus, combined studies on metagenomics and metabolomics, coupled with molecular pathways analysis, are essential to elucidate alterations in methionine metabolism and the implication for CDX2 gene downregulation during GIM progression to GC.

## 6. Conclusions

Gastric IM is an earlier precancerous lesion with the risk of neoplastic transformation to dysplasia and GC. Early detection and prognostication remain challenging because of the difficulty with the traditional endoscopic method, which is complicated by the increased rate of inappropriateness and false negativity, implying a dire need for potential clinical biomarkers. Moreover, endoscopic surgical treatment intervention have only only been considered in patients with clearly identified meta plastic lesions, which made less accessible and less effective due to a potential risk for recurrence and incidence of interval early GC. Although early prevention approaches were highly recommended through the eradication of *H. pylori*, their implementation has been varied with the regional disparities in the disease burden. Moreover, there is a lack of consensus on the study reports regarding the reversibility of GIM. Thus, currently, there is a huge unmet need for alternative diagnostic biomarkers and treatment modalities for GIM which could improve the existing strategies. Accumulated evidences indicated that metabolic alteration in amino acid metabolism, such as methionine, could play a role in predictive screening and preventive therapies to control tumorigenesis and enhance precision medicine. Previous studies indicated the occurrence of preliminary tumorigenic changes, such as a shift in the gut microbiota composition with an increasing relative abundance of Gram-positive bacteria (*lactobacillus*) over *H. pylori* in the progression of CAG to GIM toward dysplasia and GC. The relative abundance of *lactobacillus* was demonstrated to be positively correlated with dietary methionine metabolism in GC. The GIM was also identified to harbor molecular alterations that could shed light on the alteration in methionine metabolism. Moreover, study evidences also indicated the potential of upregulation in the oncogenic signaling via the PI3K/Akt/mTOR/c-MYC pathway in GIM, which could facilitate the metabolic rewiring of dietary methionine as a precursor to the biosynthesis of SAM required for transmethylation. Accordingly, a profile of an increased DNA methylation index was reported in GIM with the downregulation of several tumor suppressor genes. The downregulation of the CDX2 gene, as an intestinal-specific tumor suppressor gene, was strongly associated with colonic-type epithelial cell proliferation and GIM progression. The *CDX2* gene is downregulated through promoter region hypermethylation, which was identified to be correlated with the patients dietary history of reduced folate intake, suggesting a potential alteration in methionine metabolism. Several studies demonstrated that targeting tumor methionine addiction could limit cellular growth, proliferation, and tumor progression. As the downregulation of the *CDX2* gene and the alteration in gut microbiota were both identified as the key determinants of GC progression and treatment failure, the link to dietary methionine metabolism could be a promising target for therapeutic exploitation in GIM. Moreover, tumor cell dietary methionine metabolism renders a metabolic phenotype characterized by reduced host circulating methionine level but with a concomitant elevated level of circulating SAM that could be evaluated for a potential clinical biomarker role in the predictive screening and prognostication of GIM to GC. Thus, future integrated molecular studies are highly required to unveil the effect of the gut microbiota shifts on dietary methionine metabolism and the role of molecular alterations, such as activated signaling via the PI3K/AKT/mTOR/c-MYC pathways to facilitate metabolic rewiring to dietary methionine sources and the subsequent aberrant DNA methylation on the downregulation of tumor suppressor genes, such as the *CDX2* gene and cellular proliferation during GIM progression.

## Data Availability

Data sharing is not applicable to this article as no datasets were generated or analyzed during the current study.
